# A Possible Molecular Mechanism of Immunomodulatory Activity of Bilirubin

**DOI:** 10.1155/2013/467383

**Published:** 2013-04-09

**Authors:** Hideto Isogai, Noriaki Hirayama

**Affiliations:** Basic Medical Science and Molecular Medicine, Tokai University School of Medicine, 147 Shimokasuya, Kanagawa, Isehara 259-1143, Japan

## Abstract

Bilirubin is an endogenous product of heme degradation in mammals. Bilirubin has long been considered as a cytotoxic waste product that needs to be excreted. However, increasing evidence suggests that bilirubin possesses multiple biological activities. In particular, recent studies have shown that bilirubin should be a protective factor for several autoimmune diseases such as rheumatoid arthritis, multiple sclerosis, and systemic lupus erythematosus. Since these autoimmune diseases are closely associated with specific types of human leukocyte antigens (HLAs), we have hypothesized that bilirubin might bind to the antigenic peptide-binding groove of the HLA molecules and exert its immunosuppressive actions. In order to evaluate the hypothesis, theoretical docking studies between bilirubin and the relevant HLA molecules have been undertaken. The *in silico* studies have clearly shown that bilirubin may bind to the antigenic peptide-binding groove of the HLA molecules relevant to the autoimmune diseases with significant affinity. The bound bilirubin may block the binding of antigenic peptides to be presented to T cell receptors and lead to suppression of the autoimmune responses. Based on this hypothesis new drug discovery research for autoimmune diseases will be conducted.

## 1. Introduction

Bilirubin is an end product of heme degradation in mammals. Although bilirubin has long been considered as a cytotoxic waste product, the beneficial properties of bilirubin have been identified during the last few decades. One possible physiologic role of bilirubin is as an antioxidant [1], and most current studies on the physiological functions of bilirubin focus on this effect. Although other functions of bilirubin besides the antioxidant effects are not well documented, increasing evidence suggests that bilirubin possesses potential immunomodulatory properties. In particular, recent studies have suggested that bilirubin effectively suppresses several autoimmune diseases. Liu and colleagues have demonstrated that bilirubin possesses powerful immunomodulatory activity and suppresses experimental autoimmune encephalomyelitis (EAE) [2]. Fischman and colleagues have shown that higher serum total bilirubin levels are protective against rheumatoid arthritis (RA) based on the secondary analysis of National Health and Nutrition Examination Survey data collected between 2003 and 2006 [3]. Peng and colleagues have observed that serum bilirubin concentrations in patients with multiple sclerosis (MS) are significantly reduced [4]. Vítek and colleagues have found that serum bilirubin levels in systemic lupus erythematosus (SLE) patients are significantly lower compared to the control population [5]. One of the plausible mechanisms of the immunomodulatory activity of bilirubin would be derived from the antioxidant activity. However, the results by Liu and colleagues have clearly demonstrated that the potent immunosuppressive effects of bilirubin cannot be attributed to its antioxidant activity. They also have shown that bilirubin has a broad suppressive effect on T cell reactivity [2]. 

Considering the background above and the well-known facts that certain autoimmune diseases are closely associated with specific types of human leukocyte antigens (HLA), we have hypothesized that bilirubin might significantly interact with the relevant HLA molecules and hinder the binding of antigenic peptides that trigger the immune responses. The crystal structures of various types of HLA molecules have revealed that their three-dimensional structures are highly conserved. The suppression of autoimmune diseases by bilirubin could be due to the common interactions between the disease-associated HLA molecules and bilirubin. However, no experimental data that indicate the direct interactions between bilirubin and the HLA molecules have been found in the literature so far. As the possibility of such interactions can be explored by theoretical docking simulations with reasonable accuracy now, we have undertaken the docking simulations between bilirubin and the relevant HLA molecules.

## 2. Method

A software system MOE (Molecular Operating Environment) [6] was used throughout this study. All the calculations were performed on a DELL PC workstation T7500.

**Figure 1:**
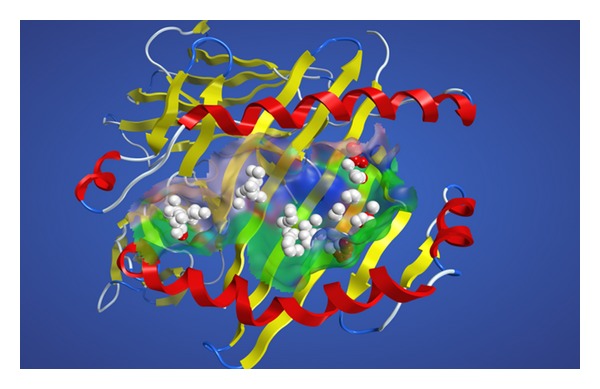
The alpha spheres calculated in the antigenic peptide-binding groove. The HLA molecules are depicted schematically. The alpha helix and the beta strand are shown in red and yellow, respectively. The white and red alpha spheres represent hydrophobic and hydrophilic positions, respectively.

Judging from the immunosuppressive effects of bilirubin, it is highly possible that bilirubin binds to the antigenic-peptide binding groove located between two *α* helices as illustrated in Figure 1. As the groove is relatively wide, we have predicted the possible binding sites of bilirubin by the use of the alpha site finder function [7] implemented in MOE. Small spheres named alpha spheres shown in Figure 1 correspond to locations of tight atomic packing at the antigenic-peptide binding groove in this case. A site where the alpha spheres are clustered is designated alpha site which is considered to be the potential binding site of small molecules. All docking simulations were undertaken by use of software ASEDock [8]. ASEDock is mainly based on two concepts. One is an ASE model, which is defined by the combination of alpha spheres generated at a concave in a protein and the excluded volumes around the concavity. The other is an ASE score, which evaluates the shape similarity between the ligand and the ASE model. The ASE score selects and refines the initial pose by maximizing the overlap between the alpha spheres and the ligand, and minimizing the overlap between the excluded volume and the ligand. Because the ASE score makes good use of the Gaussian-type function for evaluating and optimizing the overlap between the ligand and the site model, it can pose a ligand onto the docking site relatively faster and more effectively than using potential energy functions. Because the posing algorithm of ASEDock is free from any bias except for shape, it is a very robust docking method. A validation study using 59 high-quality X-ray structures of the complexes between drug-like molecules and the target proteins has demonstrated that ASEDock can faithfully reproduce experimentally determined docking modes of various drug like molecules in their target proteins [8].

The binding affinity of bilirubin to the HLA molecule was judged by a scoring function of GBVI/WSA [9] which is considered to express protein-ligand binding free energy. 

For docking validation, redocking was performed using the recently published X-ray structure of the complex between an anti-HIV drug abacavir and the HLA-B ∗ 57:01 molecule [10]. This is the only X-ray structure of the complex between a HLA molecule and a small organic molecule reported so far. The three-dimensional coordinates (3VRI) were obtained from the Protein Data Bank (PDB) [11]. The root-mean square deviation(rmsd) between nonhydrogen atoms of abacavir in the crystal and docked structures is 0.99 Å. Since prediction within rmsd of 2.0 Å is held as the passing standard, the results indicate that ASEDock is suitable for the docking simulations of the HLA-bilirubin system. The GBVI/WSA_dG value was −6.56 kcal/mol.

**Figure 2:**
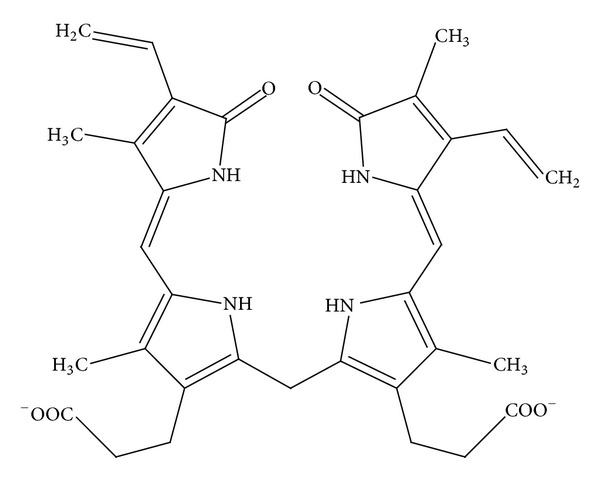
Chemical structure of bilirubin adopted in this study.

Since the 4*Z*, 15*Z*-bilirubin IX*α* form with negatively charged carboxylic groups as illustrated in Figure 2 is the predominant form of bilirubin *in vivo *[12], this isomer was adopted in this study. 

The PDB structures of 3O6F and 2WBJ were used for the HLA DR4 and DR2 molecules associated with RA and SLE, respectively. The X-ray structure of the HLA DP5 molecule associated with MS was not available when we started the present study. Therefore the 3D structure was built by homology modeling using the corresponding most-homologous HLA molecule deposited in PDB. The PDB structure of 3LQZ was selected as a template because of its high sequence identity with HLA DP5 and the high-quality structure. The homology modeling method implemented in MOE was used. The method is based on a combination of the segment-matching procedure [13] and an approach to the modeling of indels [14]. Ten models were generated by making a series of Boltzmann-weighted choices of side chain rotamers and loop conformations from a set of protein fragments selected from a library of high-resolution protein structures. An average model was energy minimized using AMBER99 force field [15].

## 3. Results and Discussion

**Figure 3:**
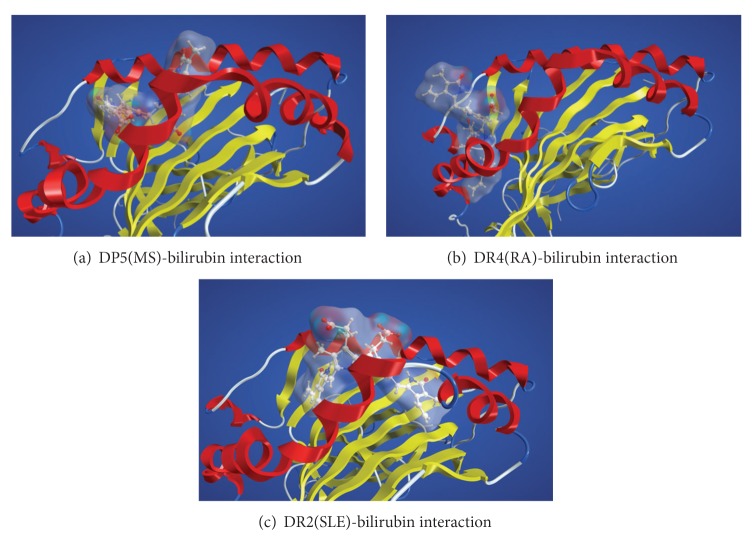
The binding modes between bilirubin and three disease-associated HLA molecules. The bilirubin molecule is shown by a ball-and-stick model with the molecular surfaces. The HLA molecules are depicted schematically. The alpha helix and beta strand are shown in red and yellow, respectively. (a) The interaction between bilirubin and the HLA DP5 molecules associated with MS (b) the interaction between bilirubin and the HLA DR4 molecules associated with RA, (c) the interaction between bilirubin and the HLA DR2 molecules associated with SLE.

The simulations have demonstrated that bilirubin should bind to the peptide-binding grooves with relatively high affinity, albeit the binding positions are somewhat different depending on the HLA molecule. The binding modes between bilirubin and the three HLA molecules are illustrated in Figure 3. Bilirubin is a relatively bulky molecule and it occludes over a large area of the peptide-binding groove. In addition, a part of the bound bilirubinprotrudes towards the space where the T cell receptor is supposed to approach to recognize the antigenic peptide. The GBVI/WSA values for the docked structures between bilirubin and the DR2, DR4, and DP5 molecules are −8.83, −8.72, and −7.02 kcal/mol, respectively. The corresponding pIC50 values are estimated to be 6.49, 6.41, and 5.16, respectively, at *T* = 298 K. The values suggest that the bound bilirubin molecules would significantly block the binding of antigenic peptides to the peptide-binding grooves leading to suppression of immunological responses. Predicted high affinities of the bilirubin molecules against the relevant HLA molecules support the above hypothesis.

This hypothesis suggests that the immunoregulatory activity of bilirubin could be derived from its binding affinity to the relevant HLA molecules. The hypothesis would foster new suspicions that different antigenic peptides might bind to the bilirubin-HLA complexes. Recently, Illing and colleagues [10] have clearly shown that binding of an anti-HIV drug abacavir at the antigen-binding groove guides the selection of new peptides. The peptides bind to the abacavir-HLA complex. Abacavir is a relatively small molecule which binds to the bottom of the antigen-binding groove. Therefore, the new endogenous peptide can bind to top of the drug-modified HLA causing adverse effect of abacavir. However, since bilirubin is much bulkier molecule than abacavir, it cannot bind to the bottom of the groove. Instead, bilirubin binds near the surface of antigen-binding groove as illustrated in Figure 3. Moreover, the protruding moiety of the bound bilirubin would prevent any peptides from further binding. This particular binding mode of bilirubin might be the reason for nonspecific suppression of several autoimmune diseases by bilirubin.

The evaluation results of our hypothesis strongly suggest that much more studies about the relationships between bilirubin and autoimmune diseases should be undertaken. The hypothesis also implies the clinical possibility of bilirubin for treatment or alleviation of particular autoimmune diseases. However, recently Khan and Poduval have demonstrated that clinically relevant concentrations of unconjugated bilirubin induce apoptosis and necrosis in immune cells by depleting cellular glutathione [16]. In addition, since there are no rich natural sources of bilirubin and it is rather expensive to synthesize it, the clinical applications of bilirubin itself do not seem to be practical. From the point of clinical applications, we must find compounds which can be effectively substituted for bilirubin. It is very interesting to start a drug discovery project to search for safer compounds which can mimic bilirubin.

## 4. Conclusions

Several studies have suggested recently that bilirubin possesses potential immunomodulatory properties. Here it is proposed that specific interactions between bilirubin and the HLA molecules associated with autoimmune diseases might contribute to the immunomodulatory activities. This hypothesis was evaluated by theoretical docking simulations. If this hypothesis turns out to be correct, it would certainly lead to the development of new drugs for treatment of autoimmune diseases for which adequate pharmacotherapy is still lacking.
